# The impact of an online educational game on breast cancer awareness among university female students, Malaysia: a pilot study

**DOI:** 10.1186/s12885-023-11427-8

**Published:** 2023-10-06

**Authors:** Jun Wey Andrew Tong, Mei Qi Hee

**Affiliations:** Department of Clinical Pharmacy, Faculty of Pharmaceutical Sciences, UCSI University Kuala Lumpur Campus, Kuala Lumpur, 56000 Malaysia

**Keywords:** Breast cancer awareness, Online educational game, Serious game, Female university student

## Abstract

**Background:**

Breast cancer is one of the world’s most prevalent cancer and the most common type of cancer in Malaysia. Interestingly, breast cancer in young women is more aggressive compared to older women and the survival rate among these groups of individuals is poor. Thus, breast cancer awareness is essential among young women as early detection is possible and treatment will be effective during which the disease is curable. Hence, the purpose of this study is to design and evaluate the impact of an educational game on breast cancer awareness among female university students in Malaysia.

**Methods:**

This is a one-group pre-and post-intervention pilot study. It was conducted in Private and public higher education institutions around Malaysia. An online education game was created and used as the intervention. A self-administered questionnaire was administered to the participants during the pre-and post-intervention test to evaluate the online educational game on breast cancer awareness.

**Results:**

A total of 52 responses were collected. The mean age of the participants was 21.98 (SD = 1.896) years. The findings showed a statistically significant median increase (p < 0.05) in breast cancer knowledge scores among participants in the post-intervention. A median increase in breast cancer knowledge score of 6 was shown when participants were exposed to the online education game (24.00) compared to before they were exposed to it (17.00).

**Conclusion:**

Using online educational games effectively raised awareness of breast cancer among university students. Online games can be used as a health educational tool to promote awareness of a topic of interest, as digital games can be accessed easily, game content can be tailored made or updated, and improve participant engagement in learning.

## Background

According to the World Health Organization (WHO), breast cancer is the world’s most prevalent cancer with a total of 7.8 million women diagnosed with breast cancer from 2015 to 2020 globally [1]. In 2020 alone, there were 2.3 million new breast cancer cases and 685,000 deaths.1 The International Agency for Research on Cancer estimates an increment of 64.2% of new breast cancer cases in Malaysia from 2020 to 2040. Despite extensive progress and effort in treatment, breast cancer remains one of the most life-threatening conditions among women including adolescent and young adults (AYAs) of age 15–39 years with increasing incidence and mortality rates [2]. Thirty per cent of cancers among AYAs are breast cancer at diagnosis. AYAs are more likely than older women with breast cancer to present with unfavourable biology (aggressive breast cancer subtypes including triple-negative [TN] or human epidermal growth factor receptor 2 [HER2]–positive) and are more likely to be found at advanced stage, when it is often more aggressive and difficult to treat, resulting in a 39% lower survival rate compared with older women [2–4].

A comprehensive review revealed that breast cancer knowledge and awareness were poor among AYAs [5]. Centers for Disease Control and Prevention (CDC) emphasized the importance of understanding their cancer risk and being proactive about their health to help to lower their risk for getting breast cancer at a young age or finding it at an early stage when treatment works best [6]. This highlights the importance of raising more awareness among the younger population.

The AYA generation has grown up using the internet and social media platforms [7]. In 2018, a Pew Research Centre survey found that 45% of AYAs are constantly online, and 97% are using social media platforms [8], which provide an opportunity for health educators to engage with AYAs through information and communication technology (ICT). Digital games that are designed to be played on a computer, video game console, mobile device, or interactive television, are often seen as a suitable platform through which ICT can create such opportunities [9]. Multiple digital educational games are published to help in disease prevention and health promotion in the areas of cancer awareness (Re-mission), improving skill and knowledge (night shift), disease prevention (COVID-19–Did You Know?), physical and mental health [10–15]. They have been recommended as an effective public action to deliver information to the public in an innovative, engaging, and entertaining way. These types of digital games are often referred to as serious games, with the use of games for training and education and have been defined as any form of interactive computer-based game software for one or multiple players to be used on any platform and that has been developed to be more than entertainment [16]. Currently, there are no studies that have been conducted to measure the impact of a digital educational game on breast cancer awareness. The purpose of this study is to investigate the use of an educational game to enhance breast cancer awareness. A digital game was developed to evaluate its impact on breast cancer awareness through a single pre/post-intervention pilot study.

## Methods

### Study design, setting and sample

A single pre/post-intervention pilot study was conducted on a convenient sample of 30 Malaysian female students from private and public higher education institutions with age ≥ 18 years old, live in Malaysia, own a laptop, and can speak, read, and write in English [17, 18]. Those who are unwilling to participate and previously participated in a similar education intervention were excluded from the study. A combination of sampling methods including snowball and convenient sampling were used in this study to recruit the participants.

An online self-administered questionnaire was distributed to the eligible participants to assess their baseline breast cancer awareness in the pre-intervention phase. Participants were asked to provide their phone number and email address for subsequent communication between the researchers and participants to ensure their full participation.

### Study instrument

A self-administered questionnaire was administered in pre-intervention and post-invention phases. The questionnaire consisted of 2 sections, including Section A: 12 socio-demographic items, Section B: 24 breast cancer knowledge items. The questionnaire was reviewed and validated by various experts from medical and academicians who have had conducted and published validated quantitative studies. An online Google form was created, and the link was disseminated to the participants through various online social platforms including WhatsApp and Facebook. The QR code to access the Google form was distributed physically to participants at the campus. They were also encouraged to share the Google form link with their classmates or friends for snowball sampling. The same questionnaire was administered before and after the intervention to access the changes in their breast cancer awareness. A digital educational game was developed as a platform-styled video game with a total of 5 levels. The main character named Emily (participant) was required to complete the game by killing a group of enemies and answering breast cancer knowledge related questions at each level. Information and keywords related to breast cancer knowledge were provided to the main character.

**Fig. 1:**
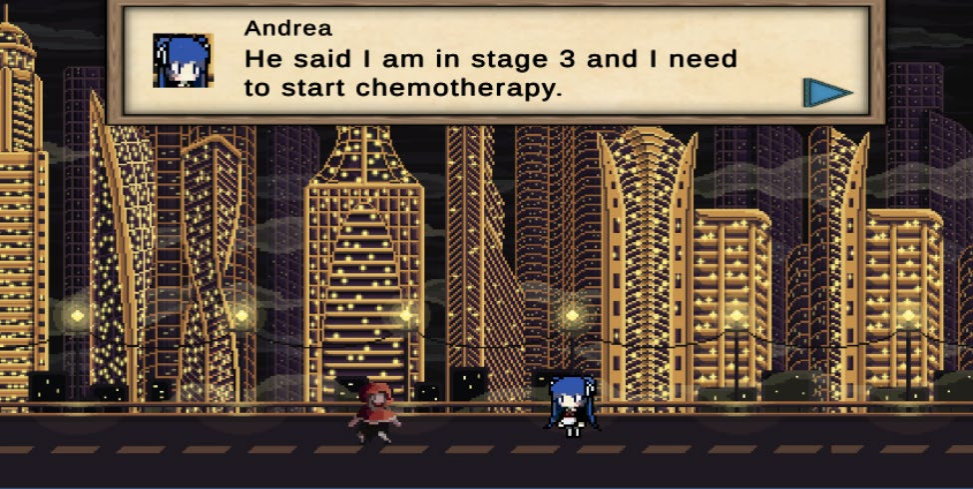
The beginning of the game

**Fig. 2:**
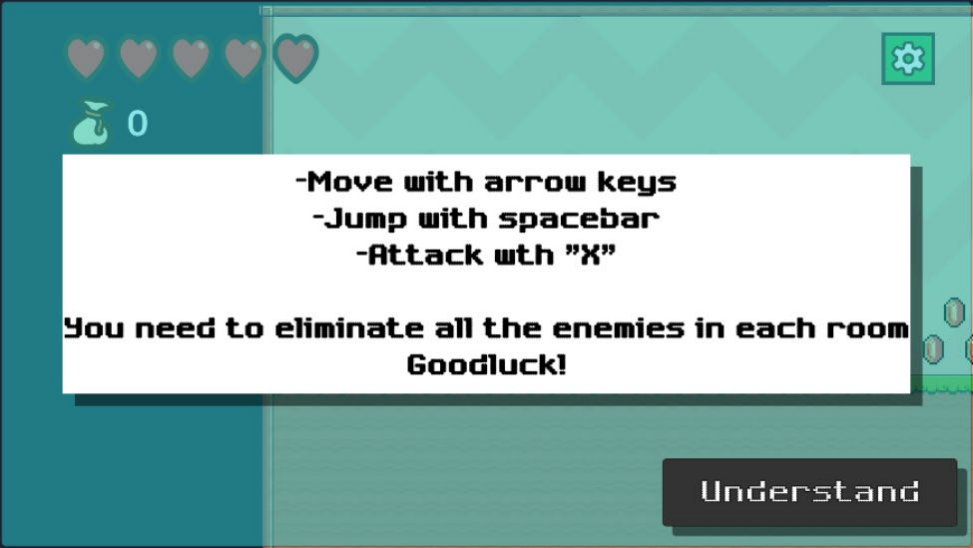
Instructions for the participant

A platform-styled video game was created using the game-creation programme “Unity” (Figs. 1, 2, 3, 4, 5 and 6). There was a total of 5 levels in the game. Emily (the participant), the primary character, was required to finish the game by killing enemies and answering breast cancer-related questions at each level. Players were given information or keywords linked to breast cancer knowledge, and they had to apply these to solve the question. Participants had to answer all the questions before proceeding to the next level and the game ended when all 5 levels were completed.

### Intervention

The game began with the participant (Emily) received a message from her friend, Andrea asking her to meet up in the town. Andrea informed Emily of her breast cancer diagnosis and treatment plan. Andrea urged Emily to get mammography, but Emily refused because she was afraid. Emily arrived home and fell asleep in her bed. That night, she had a dream about some enemies (cancer cells) attacking her.

Game-play consisted of guiding the participant (Emily) character to destroy enemies (cancer cells), for example moving, jumping, attacking and how to proceed to the next level.

**Fig. 3a:**
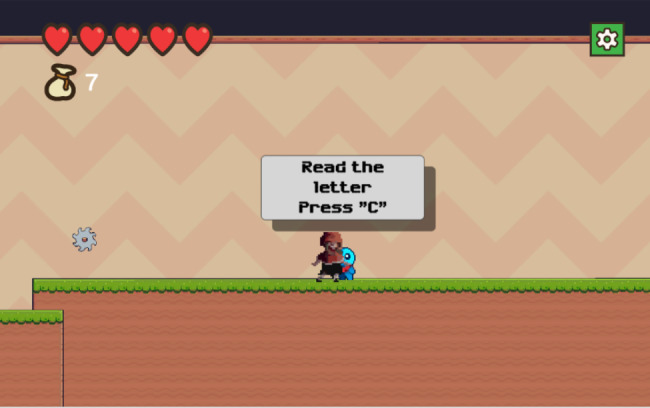
Obtain key words/information related to breast cancer

The participant can engage with the blue creature (sprite) by pressing the letter “C” on their keyboard to open a text message about breast cancer (Fig. 3b).

**Fig. 3b:**
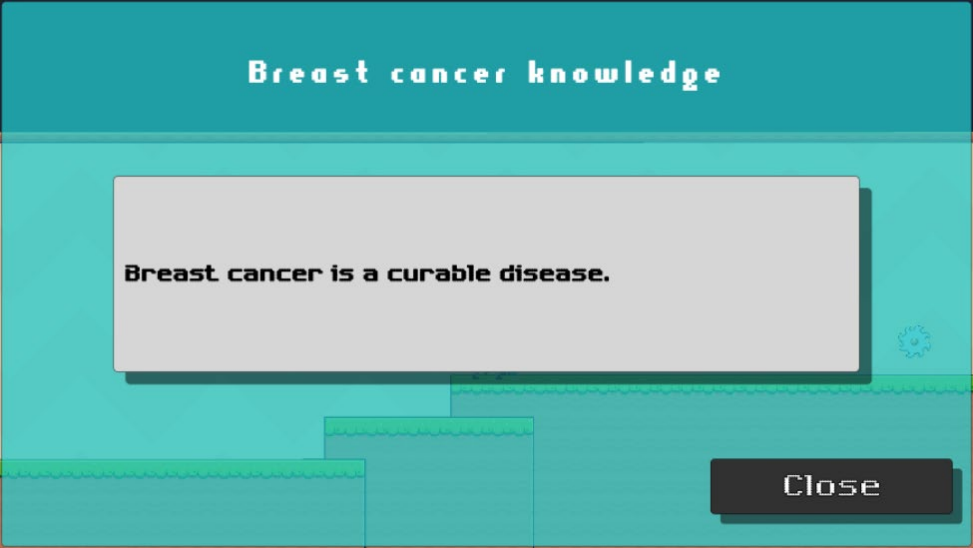
Obtain key words/information related to breast cancer

These blue creatures (sprite) can be found throughout the game, providing breast cancer information to the participant, including general knowledge, signs and symptoms, risk factors, treatment, and screening.

**Fig. 4:**
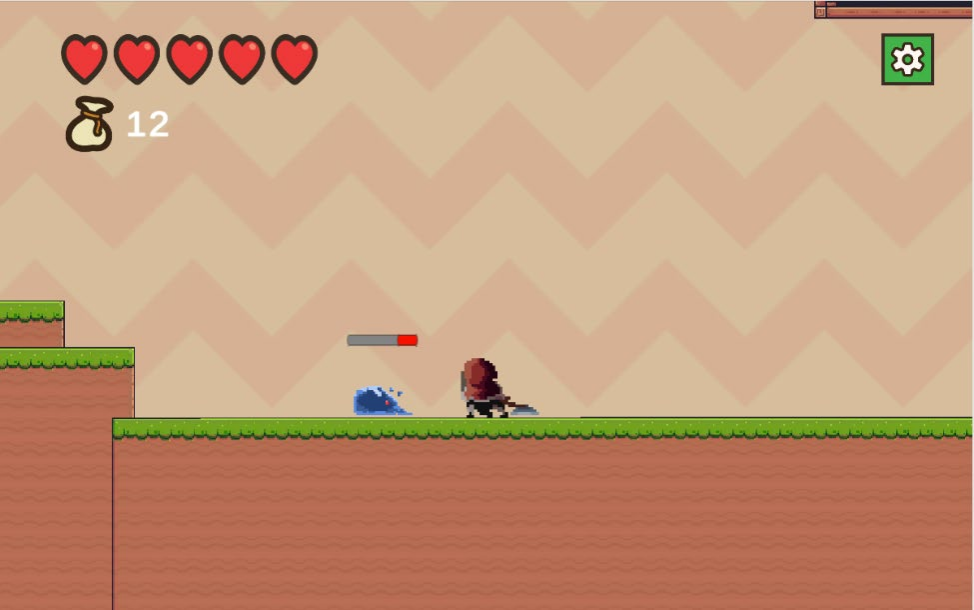
An example of enemy (cancer cells)

To advance to the next level, participant must defeat all the enemies. Enemies can inflict damage on players, which reduces one red heart (top left of the screen) per hit. Participant must restart the level (respective level) if they lose all of their red hearts.

**Fig. 5:**
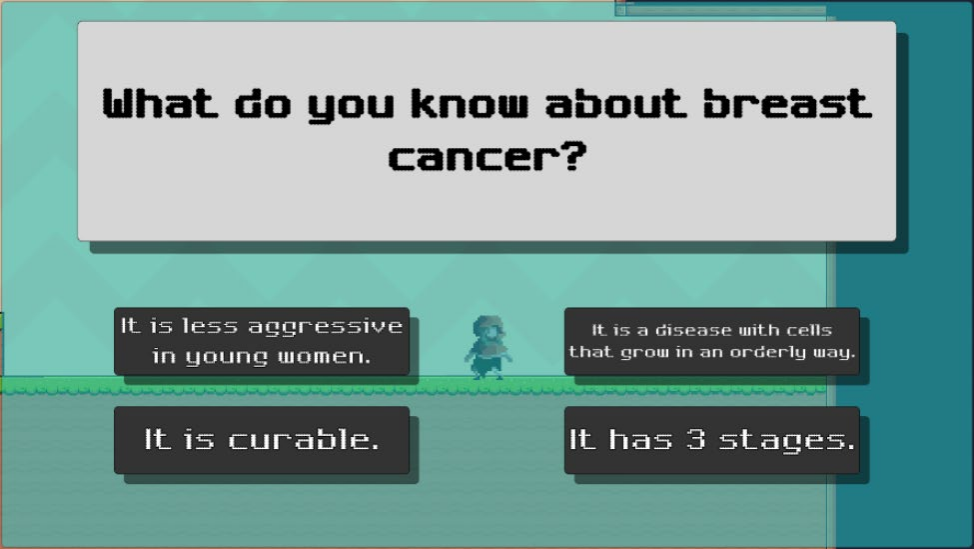
The final stage of each level

Before moving to the next level, participants had to answer all of the breast cancer-related questions. By answering correctly, participants will receive power level-ups such as increased damage inflicted to enemies. If the answer was incorrect, the correct answer will be given.

**Fig. 6:**
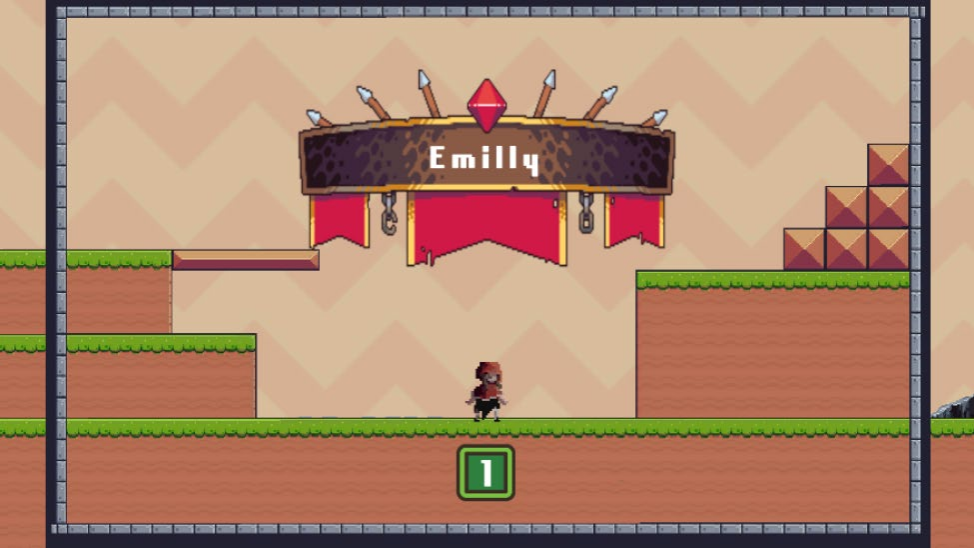
Completion of the game

The image above shows the game’s end after participant completed all 5 levels.

### Data collection

Data were collected from August to October 2022 from web and physical. An online Google form was disseminated to the participants through various online social platforms including WhatsApp and Facebook. The QR code to access the Google form was distributed physically to participants at the campus. They were also encouraged to share the Google form link with their classmates or friends who fulfilled the recruitment criteria. Once the participants completed the pre-intervention questionnaire, the digital game link and post-intervention questionnaire were shared to the participants.

### Data analysis

The Statistical Packages for the Social Sciences (SPSS) version 20 was used for the descriptive and inferential analysis of the data. Categorical data were summarized using frequencies and percentages. For knowledge items, ‘1 mark’ for correct response and ‘0’ mark for incorrect response and do not know. The data obtained were not normally distributed, a Mc-Nemar test. and Wilcoxon Signed Rank test were used to determine pre- and post-test changes. The results were expressed in median ± standard deviation (SD) and a p-value of < 0.05 was considered statistically significant.

### Ethical declarations

This study was approved by the Faculty Research and Scholar Activities (FRSA), Faculty of Pharmaceutical Science, UCSI University and Institutional Ethics Committee (IEC-2022-FPS-044), UCSI University. All methods were carried out in accordance with relevant guidelines and regulations. Written informed consent was obtained from each participant who was assured of confidentiality and anonymity before their participation. Any type of dissemination of the data was done anonymously.

### Result

#### Sociodemographic of participants

The sociodemographic characteristics of participants were presented in Table 1. A total of 75 responses were collected with 53 out of 75 completed the pre- and post-test, giving an overall response rate of 70.6%. The mean age ± SD of participants was 21.98 years ± 1.98. Most of the participants were Chinese (77.4%), followed by Malays (13.2%) and Indians (3.8%). 71.7% of participants were from private institutions and 28.3% were from the public institutions. Mostly these participants were undertaking a degree (81.1%), and 62.3% were from healthcare-related studies.

**Table 1:** Sociodemographic characteristics of participants (n = 75)

Variable (n = 53)	Frequency (n)	Percentage (%)
Age mean (SD)	21.98 (1.98)	
Age		
< 21	10	18.87
= 21 > 21	4 39	7.55 73.58
Ethnicity		
Malay	7	13.2
Chinese	41	77.4
Indian	2	3.8
Other	3	5.7
Religion		
Muslim	8	15.1
Buddhist	28	52.8
Christian	14	26.4
Hinduism	1	1.9
Other	2	3.8
Type of Education		
Public	15	28.4
Private	38	71.7
Degree of Education		
Pre-university	5	9.4
Diploma	3	5.7
Degree	43	81.1
Other	2	3.8
Discipline of study		
Healthcare related	33	62.3
Non-healthcare related	20	37.7

Table 2 describes the responses of the participants on family history of breast cancer and breast self-examination (BSE). From the total sample collected, many of the participants reported that they do not have a family history of breast cancer (88.7%) and their relationship with breast cancer patients was second-degree relative. 77.4% participants were aware of BSE, however about half of them (45.3%) did not practice BSE.

**Table 2:** Response on family history of breast cancer and BSE

Variable	Frequency (n)	Percentage (%)
Family history of breast cancer		
Yes	6	11.3
No	47	88.7
Have you heard of Breast Self-examination (BSE)?		
Yes	41	77.4
No	12	22.6
Do you practice BSE?		
Yes	17	41.46
No	24	58.53
Age of first practicing BSE		
Before 20 years old	14	26.4
20 to 30 years old	3	5.7

Tables 3 and 4 summarize the participants’ responses pre- and post-intervention on breast cancer knowledge. Most of the breast cancer knowledge items showed a significant improvement after intervention. Some items had an intermediate or high score in the pre-intervention study (such as sign and symptoms of breast cancer), the improvements after intervention were still drastically. The item with the lowest score and minimal improvement was reported for never breastfeeding (risk factor).

The majority of the breast cancer knowledge items improved significantly (p < 0.05) following intervention especially item no 2 (35.85% vs. 96.23%) under general knowledge section; item no 2 (49.06% vs. 92.45%) and item no 3 (30.19% vs. 66.04%) under risk factors section; item 4 (56.60% vs. 98.11%), item 7 (43.40% vs. 84.91%) and item 8 (35.95% vs. 86.79%) under breast cancer screening and treatment section. Some of the items had intermediate or high scores in the pre-intervention trial (such as signs and symptoms of breast cancer), the changes after intervention were still significant. Never breastfeeding (risk factor) received the lowest score and showed the least improvement.

**Table 3:** Comparison of pre- and post-intervention on breast cancer knowledge using Mc Nemar test

Variable	Pre-intervention^a^		Post-intervention^a^		P-value*
	Yes n (%)	No/I don’t know n (%)	Yes n (%)	No/I don’t know n (%)	
General Knowledge					
“Breast cancer happens when cells begin to grow out of control”	48 (90.56)	5 (9.44)	53 (100)	0	0.63
“Breast cancer presents in young women is more aggressive than those of older women”	19 (35.85)	34 (64.15)	51 (96.23)	2 (3.77)	0.00
“Breast Cancer is a curable disease”	36 (67.92)	17 (32.08)	53 (100)	0	0.00
“ There are 5 stages of breast cancer: stage 0 is non-spreading breast cancer, and stage 1 through stage 4 is spreading breast cancer”	36 (67.92)	17 (32.08)	52 (98.11)	1 (1.89)	0.00
Risk Factors					
Age	41 (77.36)	12 (22.64)	53 (100)	0	0.00
Family History and personal history of breast cancer	46 (86.79)	7 (13.21)	53 (100)	0	0.16
Early menstruation (< age 12) and late menopause (> age 55)	26 (49.06)	27 (50.94)	49 (92.45)	4 (7.55)	0.00
Never Breastfeeding	16 (30.19)	37 (69.81)	35 (66.04)	18 (33.96)	0.00
Long term use of hormone replacement therapy	35 (66.04)	18 (33.96)	51 (96.23)	2 (3.77)	0.00
Lifestyle such as smoking, alcohol use and physically inactive	48 (90.56)	5 (9.44)	52 (98.11)	1 (1.89)	0.125
Sign and symptoms					
Change in the shape and size of the breast or nipple	45 (84.91)	8 (15.09)	53 (100)	0	0.08
Lump or thickening in the breast, armpit area and collarbone area	45 (84.91)	8 (15.09)	53 (100)	0	0.08
Unusual pain or tenderness in the breast or armpit	45 (84.91)	8 (15.09)	52 (98.11)	1 (1.89)	0.39
Dimpling, puckering or scaling of the breast skin	39 (73.58)	14 (26.42)	52 (98.11)	1 (1.89)	0.00
Abnormal discharge from the nipple including watery, milky, yellow fluid and blood	42 (79.25)	11 (20.75)	52 (98.11)	1 (1.89)	0.02
Breast cancer screening and treatment					
Breast screening can help to detect breast cancer early and avoid delays in care	51 (96.23)	2 (3.77)	53 (100)	0	0.50
Breast self-exam can increase breast cancer awareness	52 (98.11)	1 (1.89)	53 (100)	0	1.00
Breast cancer screening includes mammography, magnetic resonance imaging (MRI) and breast ultrasound	45 (84.91)	8 (15.09)	53 (100)	0	0.08
Mammography is recommended for women aged at least 50 years old every 1–2 years	30 (56.60)	23 (43.40)	52 (98.11)	1 (1.89)	0.00
Surgery	49 (92.45)	4 (7.55)	52 (98.11)	1 (1.89)	0.25
Chemotherapy	44 (83.02)	9 (16.98)	52 (98.11)	1 (1.89)	0.08
Hormonal therapy	23 (43.40)	30 (56.60)	45 (84.91)	8 (15.09)	0.00
Biological therapy	19 (35.95)	34 (64.15)	46 (86.79)	7 (13.21)	0.00
Radiation therapy	34 (64.15)	19 (35.85)	49 (92.45)	4 (7.55)	0.00

Note: a = Mc Nemar tets, * P < 0.05, significant

A Wilcoxon signed-rank test showed a statistically significant median increase in breast cancer awareness score post-intervention (24.00) compared to pre-intervention (17.00), z = -6.101, p < 0.05.

**Table 4:** Comparison of pre- and post-intervention on breast cancer knowledge using Wilcoxon Signed Rank test

	N	Mean			Std. Deviation	Minimum		Maximum
Pre-intervention	53	17.26			4.096	8.00		24.00
Post-intervention	53	23.00			1.481	17.00		24.00
Pre-intervention				Post-intervention			Differences	
17.0000				24.00			-6.00	
z			-6.10					
Asymp sign. (2-tailed)			0.000					

**Table 5:** Comparison of students from healthcare related studies and students from non-healthcare related studies (Response on family history of breast cancer and BSE)

Discipline of study/Variable	Healthcare related		Non-healthcare related	
	Yes n (%)	No/I don’t know n (%)	Yes n (%)	No/I don’t know n (%)
Family History of breast cancer	3 (9.09)	30 (90.91)	3 (15)	17 (85)
Have you heard of Breast Self-examination (BSE)?	30 (90.91)	3 (9.09)	11 (55)	9 (45)
Do you practice BSE	10 (30.30)	20 (60.61)	7 (35)	4 (20)

Table 5 demonstrates the differences in responses to a family history of breast cancer and BSE between healthcare and non-healthcare students. The majority of the healthcare students had heard about BSE. However, nearly half of the students in non-healthcare disciplines have never heard of BSE.

**Table 6:** Comparison of pre- and post-intervention on breast cancer knowledge between healthcare and non-healthcare students using Mc Nemar test

Discipline of study/Variable	Non-healthcare related (n = 20)				P-value*	Healthcare related (n = 33)				P-value*
	Pre-intervention^a^		Post-intervention^a^			Pre-intervention^a^		Post-intervention^a^		
	Yes n (%)	No/I don’t know n (%)	Yes n (%)	No/I don’t know n (%)		Yes n (%)	No/I don’t know n (%)	Yes n (%)	No/I don’t know n (%)	
General Knowledge										
“Breast cancer happens when cells begin to grow out of control”	17 (85)	3 (15)	20 (100)	0	0.250	31 (93.94)	2 (6.06)	33 (100)	0	0.500
“Breast cancer presents in young women is more aggressive than those of older women”	7 (25)	13 (65)	19 (95)	1 (5)	0.000	12 (36.36)	21 (63.64)	32 (96.97)	1 (3.03)	0.000
“Breast Cancer is a curable disease”	10 (50)	10 (50)	20 (100)	0	0.002	26 (78.79)	7 (21.21)	33 (100)	0	0.016
“ There are 5 stages of breast cancer: stage 0 is non-spreading breast cancer, and stage 1 through stage 4 is spreading breast cancer”	12 (60)	8 (40)	20 (100)	0	0.008	24 (72.73)	9 (27.27)	32 (96.97)	1 (3.03)	0.008
Risk Factors										
Age	12 (60)	8 (40)	20 (100)	0	0.008	29 (87.88)	4 (12.12)	33 (100)	0	0.125
Family History and personal history of breast cancer	16 (80)	4 (20)	20 (100)	0	0.125	30 (90.91)	3 (9.09)	33 (100)	0	0.250
Early menstruation (< age 12) and late menopause (> age 55)	8 (40)	12 (60)	18 (90)	2 (10)	0.002	18 (54.54)	15 (45.45)	31 (93.94)	2 (6.06)	0.000
Never Breastfeeding	4 (20)	16 (60)	11 (55)	9 (45)	0.039	12 (36.36)	21 (63.64)	24 (72.73)	9 (27.27)	0.000
Long term use of hormone replacement therapy	11 (55)	9 (45)	20 (100)	0	0.004	24 (72.73)	9 (27.27)	31 (93.94)	2 (6.06)	0.016
Lifestyle such as smoking, alcohol use and physically inactive	17 (85)	3 (15)	20 (100)	0	0.250	31 (93.94)	2 (6.06)	32 (96.97)	1 (3.03)	1.000
Sign and symptoms										
Change in the shape and size of the breast or nipple	16 (60)	4 (20)	20 (100)	0	0.125	29 (87.88)	4 (12.12)	33 (100)	0	0.125
Lump or thickening in the breast, armpit area and collarbone area	15 (75)	5 (25)	20 (100)	0	0.063	30 (90.91)	3 (9.09)	33 (100)	0	0.250
Unusual pain or tenderness in the breast or armpit	16 (60)	4 (20)	19 (95)	1 (5)	0.375	29 (87.88)	4 (12.12)	33 (100)	0	0.125
Dimpling, puckering or scaling of the breast skin	13 (65)	7 (35)	20 (100)	0	0.016	26 (78.79)	7 (21.21)	32 (96.97)	1 (3.03)	0.031
Abnormal discharge from the nipple including watery, milky, yellow fluid and blood	15 (75)	5 (25)	20 (100)	0	0.063	27 (81.82)	6 (18.18)	32 (96.97)	1 (3.03)	0.063
Breast cancer screening and treatment										
Breast screening can help to detect breast cancer early and avoid delays in care	20 (100)	0	20 (100)	0	1.000	31 (93.94)	2 (6.06)	33 (100)	0	0.500
Breast self-exam can increase breast cancer awareness	20 (100)	0	20 (100)	0	1.000	32 (96.97)	1 (3.03)	33 (100)	0	1.000
Breast cancer screening includes mammography, magnetic resonance imaging (MRI) and breast ultrasound	17 (85)	3 (15)	20 (100)	0	0.250	28 (84.85)	5 (15.15)	33 (100)	0	0.063
Mammography is recommended for women aged at least 50 years old every 1–2 years	11 (55)	9 (45)	19 (95)	1 (5)	0.008	19 (57.58)	14 (42.42)	33 (100)	0	0.000
Surgery	19 (95)	1 (5)	20 (100)	0	1.00	30 (90.91)	3 (9.09)	32 (96.97)	1 (3.03)	0.500
Chemotherapy	15 (75)	5 (25)	20 (100)	0	0.063	29 (87.88)	4 (12.12)	32 (96.97)	1 (3.03)	0.250
Hormonal therapy	4 (20)	16 (80)	18 (90)	2 (10)	0.000	19 (57.58)	14 (42.42)	27 (81.82)	6 (18.18)	0.008
Biological therapy	4 (20)	16 (80)	17 (85)	3 (15)	0.00	15 (45.45)	18 (54.55)	29 (87.88)	4 (12.12)	0.001
Radiation therapy	10 (50)	10 (50)	19 (95)	1 (5)	0.004	24 (72.73)	9 (27.27)	30 (90.91)	3 (9.09)	0.031

Note: a = Mc Nemar tets, * P < 0.05, significant

Table 6 shows healthcare students generally have higher awareness than non-healthcare students for items such as general knowledge of breast cancer, screening, and treatment.

## Discussion

Breast cancer is the most common type of cancer among females and the second leading cause of cancer death among females. Young women who are diagnosed with breast cancer tend to have a poorer survival rate (prognosis) due to the more aggressive nature of breast cancer compared to the ones in older women [19–22]. Poor prognosis in younger women may be due to the presentation at an advanced stage at diagnosis, aggressive pathological characteristics, and a higher rate of recurrence [23, 24]. According to the Malaysia Clinical Practice Guideline 2019, it is recommended for women aged 50–74 years in the general population to perform mammography screening biennially [25]. The age at which to perform breast screening and the number of screenings depends on high-risk factors or genetic variants. According to the guideline, the earliest age for breast screening is 30 for individuals with high-risk factors (mammography) or carriers of pathogenic variants (MRI annually) [25]. This presentation at an advanced stage of diagnosis can be due to a lack of emphasis on screening for young women or a lack of suspicion or awareness among the youth. Early detection and treatment of breast cancer are important, as the disease is curable before it reaches an advanced stage [26]. The earlier the detection, the higher the chance of survival because receiving treatment early to better outcomes with less complexity and lower cost [25].

The use of video games other than for the sole purpose of entertainment is called a ‘serious game’ [27]. A serious game can be developed for education, advertisement, improving lifestyles, healthcare and many other topics that need to be addressed. The use of serious games can be a beneficial alternative to the traditional learning method. There were several studies designed and developed games for cancer prevention education, raising breast cancer perception and information-seeking behaviour for instance Re-mission and night shift [28, 29]. The creativity involved in making a serious game is limitless, and developers can make an educational game that is tailored to their target participants. Consequently, a serious game’s benefit is that it imparts knowledge in a fun and engaging manner. Additionally, if a person owns digital devices, such as a smartphone, a personal computer, or a tablet, which are connected to the internet, they can access video games whenever and wherever they choose [30, 31]. Malaysia now has a growing population of users of electronic devices, including a growing number of mobile, laptop, and desktop users. As a result, video games can be an effective medium for disseminating health information [32, 33].

The results of the present study indicated that the digital education game significantly improved breast cancer awareness among young females, including their general knowledge, risk factors, signs and symptoms, and screening and treatment of breast cancer. According to our pre-intervention results, early menstruation before the age of 12, late menopause after the age of 55, use of birth control pill, not breastfeeding, and nulliparity are the less well-known risk factors, in comparison to factors like age, family history of breast cancer, and an unhealthy lifestyle. The findings were consistent with the previous studies conducted in the south of Peninsular Malaysia and Shah Alam [28, 34, 35]. The participants were able to recognize common symptoms of breast cancer, but only a few of them knew that dimpling, puckering or scaling of the breast skin (73.58%) are the other possible symptoms of breast cancer. Nearly half of the participants demonstrated poor knowledge when questioned if “mammography is indicated for women aged at least 50 years old every 1–2 years.”; breast cancer treatment options such as biological therapy and hormonal therapy. The Malaysian Clinical Practice Guidelines recommend mammographic screening begins at aged 50 in women without any identifiable risk factors [25], therefore, this explains why many young participants in our study were unaware of the recommended age for starting breast cancer screening. The same findings were reported by Lee WN et al., 2021 [36]. Newer treatments like hormonal therapy and biological therapy were less well-known among participants than single, older treatments such as surgery, radiation, and chemotherapy. This could be explained by their lack of exposure to breast cancer-related information and the fact that they are not the primary target of breast cancer awareness campaigns. Their knowledge on these items was greatly improved after the intervention. When compared to non-healthcare students, healthcare students are more aware of general knowledge, sign and symptom, screening, and treatment of breast cancer. As shown in Tables 6, both healthcare and non-healthcare participants significantly improved their breast cancer awareness after the intervention. However, the number of non-healthcare students is lower than the number of healthcare students, hence further research is required to investigate the effects of educational game on breast cancer awareness.

Overall, the participants provided positive comments, claiming that the digital educational game was an innovative tool to raise their level of health awareness and that it encouraged them to learn more. The outcome was consistent with past studies [30, 31, 37] in which it was established that learning was more engaging for the participants. Digital educational games can be a very useful tool for public, especially AYA generations, with their ease of updating game content and accessibility, entertainment value, and ability to construct improbable scenarios for learning reasons [30, 31]. Although the game was successful in spreading awareness of breast cancer, it is still unclear whether the participants would retain the knowledge. For instance, many participants learned that breast cancer symptoms in young women are more aggressive than those of older women, but it is uncertain whether their knowledge will affect their future behaviour. Further studies are required for the information retention and understanding.

The biggest drawback of the current version of digital educational game is that it is difficult for participants to use the “fighting” system. The game can be improved further in a number of ways, including (1) expanding the gameplay’s mechanics such as extension of the range of attack, smooth action, and movement, to make it easier for players to participate; (2) making it mobile devices compatible to improve its convenience and playability; (3) adding game mechanics like challenges [28]; and (4) customization of characters and “levelling up” to make the gameplay entertaining and informative [38].

## Conclusion

The previous studies showed that young women have limited breast cancer knowledge. With the high mortality rate and the fact that there is currently no prescribed breast cancer screening program for AYA women without known risk factors for the disease, it is crucial for young women to learn and understand their breast cancer risk and to be proactive about their health to lower their risk of getting breast cancer. The results of the current study show that gamification can enhance AYAs’ learning motivation and experience with breast cancer. With the advancement of educational technology and its convenience, educational games are becoming more and more popular, and further research is therefore recommended in Malaysia to improve the content of the digital game and its features on breast cancer. In conclusion, gamification can enhance breast cancer awareness among AYAs.

## Data Availability

The datasets generated or analysed during this study are included in this article.
